# External validation of the detection of indicators and vulnerabilities for emergency room trips (DIVERT) scale: a retrospective cohort study

**DOI:** 10.1186/s12877-020-01816-0

**Published:** 2020-10-20

**Authors:** Fabrice I. Mowbray, Aaron Jones, Connie Schumacher, John Hirdes, Andrew P. Costa

**Affiliations:** 1 Department of Health Research Methods, Evidence and Impact, McMaster University, 1280 Main St W, Hamilton, Ontario L8S 4K1 Canada; 2 Department of Nursing, Brock University, 1812 Sir Isaac Brock Way, St. Catherines, Ontario L2S 3A1 Canada; 3 School of Public Health and Health Systems, University of Waterloo, 200 University Avenue West, Waterloo, Ontario N2L 3E9 Canada

**Keywords:** Home care, Emergency department, Geriatrics, DIVERT

## Abstract

**Background:**

The Detection of Indicators and Vulnerabilities of Emergency Room Trips (DIVERT) scale was developed to classify and estimate the risk of emergency department (ED) use among home care clients. The objective of this study was to externally validate the DIVERT scale in a secondary population of home care clients.

**Methods:**

We conducted a retrospective cohort study, linking data from the Home Care Reporting System and the National Ambulatory Care Reporting System. Data were collected on older long-stay home care clients who received a RAI Home Care (RAI-HC) assessment. Data were collected for home care clients in the Canadian provinces of Ontario and Alberta, as well as in the cities of Winnipeg, Manitoba and Whitehorse, Yukon Territories between April 1, 2011 and September 30, 2014. The DIVERT scale was originally derived from the items of the RAI-HC through the use of recursive partitioning informed by a multinational clinical panel. This scale is currently implemented alongside the RAI-HC in provinces across Canada. The primary outcome of this study was ED visitation within 6 months of a RAI-HC assessment.

**Results:**

The cohort contained 1,001,133 home care clients. The vast majority of cases received services in Ontario (88%), followed by Alberta (8%), Winnipeg (4%), and Whitehorse (< 1%). Across the four cohorts, the DIVERT scale demonstrated similar discriminative ability to the original validation work for all outcomes during the six-month follow-up: ED visitation (AUC = 0.617–0.647), two or more ED visits (AUC = 0.628–0.634) and hospital admission (AUC = 0.617–0.664).

**Conclusions:**

The findings of this study support the external validity of the DIVERT scale. More specifically, the predictive accuracy of the DIVERT scale from the original work was similar to the accuracy demonstrated within a new cohort, created from different geographical regions and time periods.

## Background

Emergency departments (ED) are a common access point for older adults in search of medical attention [1, 2]. Older adults often present to the ED with extensive medical and psychosocial histories, increasing their risk for functional decline, readmission, and death post-discharge [3]. The time pressures and high client volumes in the ED often hinder emergency clinicians from providing comprehensive geriatric assessments and chronic disease management [4, 5]. To better support the needs of older adults, clinicians, researchers and policymakers have placed a greater emphasis on improving community-based disease management and service integration in an attempt to prevent avoidable ED visitation [6].

Approximately one-quarter of older adults in Canada are receiving home care services, with the rate of home care enrollment increasing with age [7, 8]. Older home care clients are a medically complex cohort with relatively poor access to effective chronic disease management. As a result, older home care clients visit the ED at approximately twice the rate of long-term care residents and autonomous older adults living in a private dwelling [9]. Prior work has demonstrated the utility of prognostic tools and home-based services in supporting the identification of community-dwelling older adults at risk for ED visitation [10–13].

Costa et al. developed and validated a prognostic case-finding tool known as the Detection of Indicators and Vulnerabilities of Emergency Room Trips (DIVERT) scale [14]. The purpose of this prognostic model is to estimate and classify the risk of ED use among home care clients so that health care systems can better identify clients who may benefit from additional chronic disease management services in the community [14]. Advanced knowledge of ED visitation can be used to stratify the need, type and frequency of home care services at a population level to mitigate the risk of unnecessary ED visitation. Several organizing bodies have recommended the implementation of the DIVERT scale during the provision of home care services [14, 15], as the scale supports an organized population-level response to community-based chronic disease management needs [15, 16]. Further, the DIVERT scale is being utilized as a prognostic model in an ongoing pragmatic cluster randomized controlled trial that aims to determine the efficacy of a cardiorespiratory disease management models in preventing or postponing future ED admissions in home care clients [17].

The DIVERT scale was developed and internally validated using a single hold-out sample. External validation using a separate and exclusive cohort is necessary to demonstrate the stability of model estimates in new patient cohorts [18, 19]. We set out to externally validate the DIVERT scale, across multiple jurisdictions, provinces and during a different time. Given the population-level predictions and face validity of the DIVERT scale, we hypothesized that the scale would provide similar performance in a new and unexamined cohort.

## Methods

### Study design

We conducted a population-based retrospective cohort study of home care clients in the provinces of Ontario and Alberta, and in the regions surrounding Winnipeg, Manitoba and Whitehorse, Yukon.

### Data sources

We linked multiple de-identified administrative health databases to construct our cohort. Home care clinical assessment data were extracted from the Home Care Reporting System. This national database contains demographic, clinical, functional and service utilization information on publicly funded home care clients in Canada. ED utilization data were extracted from the National Ambulatory Care Reporting System, which houses comprehensive population-level data on hospital and community-based ambulatory care visits in Canada. The databases used in this study are routinely checked for validity and have been used extensively in health services research [18–22]. The Hamilton Integrated Research Ethics Board granted ethics approval for this study. No additional administrative permission or licensure was required for this project.

### Participants

Home care clients in Canada are periodically assessed using the Resident Assessment Instrument for Home Care (RAH-HC). We created a retrospective cohort of all RAI-HC assessments completed between April 1, 2011 and September 30, 2014. Data were accessed on clients in the provinces of Ontario and Alberta, as well as in the Winnipeg Regional Health Authority in Manitoba and the Whitehorse census subdivision of the Yukon Territory. The cohorts in Manitoba and Yukon were restricted to areas surrounding the cities of Winnipeg and Whitehouse due to limitations in the coverage of the National Ambulatory Care Reporting System (NACRS). The RAI-HC assessments in the cohort were linked to ED records in the NACRS dataset to identify all ED visits within 6 months of the assessment date.

### Measurement

**Fig. 1:**
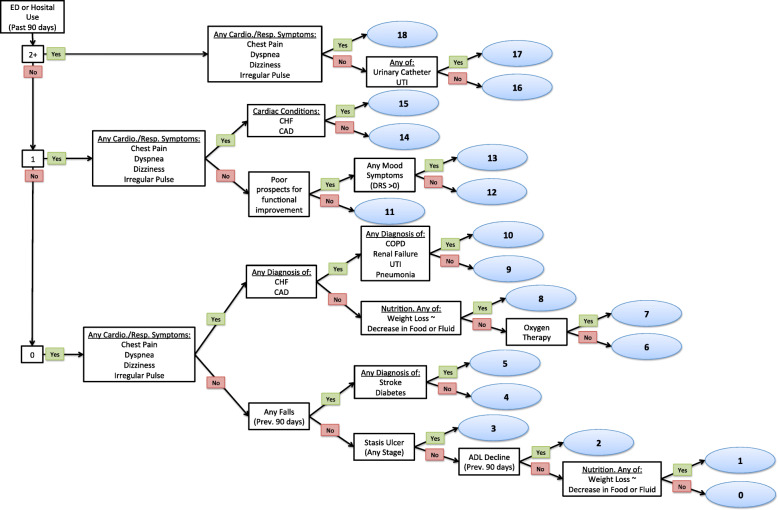
The DIVERT Scale

The DIVERT scale is a prognostic model developed using a classification and regression tree and recursive partitioning methods. Assessment items of the RAI-HC were considered as candidate predictors for model derivation. The RAI-HC is a comprehensive clinical assessment of over 250 items that have demonstrated validity and reliability in documenting the domains of function, health, social support and health service use [22, 23]. The RAI-HC is currently used during standardized home care assessments in most Canadian provinces and territories, half of the United States, and in many countries around the world including Estonia, Finland, Hong Kong, Iceland, Ireland, Italy, Japan, the Netherlands, New Zealand, Singapore, Spain and Switzerland. At this time, the DIVERT scale is a standard measure within the RAI-HC assessment. Figure 1 displays the DIVERT scale and the predictors used in discriminating patient risk for ED visitation.

### Outcome measure

The primary outcome of this study was an ED visit within 6 months after a RAI-HC assessment date. Secondary outcomes include: (a) two or more ED visits within 6 months of a RAI-HC assessment and (b) any ED visits that resulted in hospital admission. Data were censored at the date of death. All outcomes were selected to parallel the figures in the original derivation study [14]. A six-month follow-up period was chosen to reflect the contemporary home care assessment intervals.

### Data analysis

The DIVERT scale was derived and validated using home care assessment data linked to ED records from Ontario and Winnipeg between 2006 and 2010 [14]. Our examination of data from Ontario, Alberta, Winnipeg, and Whitehorse between 2011 and 2014 enables us to test the scale’s external validity within the two original regions, during a different time. We measured the discriminative ability of the DIVERT scale using the area under the receiver operating curve (AUC), or *c*-statistic. The AUC is the area under the curve created by plotting sensitivity against 1-specificity at various thresholds and is a common measure of the discriminative ability. Within each region, we calculated the AUC of the DIVERT scale for each outcome. All analysis was performed using SAS/STAT 13.1.

## Results

### Sample characteristics

Within the four regions of our study, we identified 1,001,133 RAI-HC assessed cases occurring between April 1, 2011 and September 30, 2014. The vast majority of cases were in Ontario (88%), followed by Alberta (8%), Winnipeg (4%) and Whitehorse (< 1%). A descriptive profile of the home care clients within each region can be found in Table 1. Overall, the home care clients in our sample were predominantly female, with a mean age of approximately 79.

**Table 1:** Sample Characteristics Across Canadian Provinces and Regions

Client Characteristics	Ontario	Alberta	Winnipeg	Whitehorse
	***n*** = 877,696	***n*** = 80,427	***n*** = 42,583	***n*** = 427
**Demographic Characteristics**				
Age (Mean)	78.2	79.4	78.8	74.7
Sex (Female)	64%	64%	67%	61%
**Health Characteristics**				
Activities of Daily Living				
Independent	46%	59%	64%	77%
Supervision/Limited Assistance	31%	23%	24%	15%
Extensive Assistance/ Dependent	23%	18%	12%	7%
Cognitive Impairment				
Intact	28%	36%	42%	48%
Borderline / Mild impairment	53%	46%	46%	41%
Moderate/Severe impairment	19%	18%	12%	11%
Depression Rating Scale				
0	53%	59%	68%	52%
1–2	26%	23%	22%	26%
3+	21%	18%	10%	21%
Poor Self-Reported Health	22%	16%	20%	26%
Fall in last 90 Days	39%	31%	29%	38%
Dyspnea	28%	18%	24%	29%
Bladder Incontinence	29%	31%	30%	21%
Wandering	3%	6%	2%	2%
Aggressive Behaviour	11%	15%	6%	8%
Frailty Index	0.24	0.21	0.18	0.19
Number of Medications (Mean)	7.4	7.5	6.8	6.9
**Informal Caregiver Status**				
Live-in caregiver	51%	35%	38%	30%
Caregiver express distress	24%	10%	11%	12%
Informal care hours per day (Mean)	20.0	16.1	13.2	13.4
**DIVERT Scale**				
1 (least risk)	16%	27%	25%	22%
2	28%	22%	29%	23%
3	18%	16%	19%	14%
4	21%	17%	16%	21%
5	11%	10%	7%	10%
6 (most risk)	7%	8%	4%	10%

Home care clients in Ontario had the highest level of physical and cognitive impairment, while clients in Whitehorse had the lowest. Home care clients in Ontario were also more likely to have a live-in caregiver and were more likely to have informal caregivers who express distress. However, clients in Whitehorse were the most likely to rate their health as poor and had the highest proportion of clients scoring three or higher on the Depression Rating Scale.

### External validation

The proportion of outcomes and the discriminative ability of the DIVERT scale across all regions are displayed in Table 2. For the primary outcome of ED visitation, the AUCs of the DIVERT Scale within Ontario, Alberta, and Winnipeg are very similar to one another (0.617–0.624) and the original validation work (0.62). The AUC for the Whitehorse cohort was slightly higher for this outcome (0.647).

**Table 2:** Outcome Proportion and Discriminative Ability of the DIVERT Scale

DIVERT	Ontario	Alberta	Winnipeg	Whitehorse
**Outcome: Any ED visit within 6 months**				
1	31%	35%	28%	34%
2	39%	42%	38%	46%
3	45%	48%	42%	46%
4	51%	52%	49%	62%
5	59%	61%	59%	66%
6	67%	66%	68%	75%
AUC	0.614	0.611	0.618	0.647
**Outcome: Any ED visit within 6 months of Hospital Admission**				
1	14%	16%	11%	4%
2	19%	22%	18%	20%
3	24%	27%	21%	20%
4	29%	31%	26%	27%
5	36%	36%	34%	27%
6	42%	41%	39%	37%
AUC	0.624	0.617	0.624	0.664
**Outcome: Two or more ED visits within 6 months of home care assessment**				
1	12%	15%	10%	16%
2	16%	19%	14%	18%
3	20%	23%	18%	19%
4	25%	27%	23%	33%
5	33%	36%	30%	39%
6	42%	43%	41%	40%
AUC	0.629	0.628	0.634	0.633

For the secondary outcome of two or more ED visits in the 6 months following a RAI-HC assessment, the AUCs within all four regions were similar to one another (0.628–0.634) and the original validation work (0.63). For the outcome examining hospital admission, the AUCs within Ontario, Alberta and Winnipeg were again comparable to one another (0.617–0.624), while the AUC for Whitehorse residents was slightly higher (0.664). Supplemental measures of predictive accuracy (e.g., sensitivity, specificity, etc.) were similar to the findings reported in the original validation paper.

## Discussion

### Important findings

Our study provides external validation of the DIVERT scale and demonstrates that the scales discriminative ability is generalizable to external populations in new geographic regions (Winnipeg, Manitoba and Whitehorse, Yukon Territories). Furthermore, we demonstrated that the performance of the DIVERT scale is robust to temporal changes. The DIVERT scale was able to adequately predict ED visitation, multiple ED visits and hospitalization in a sample of Canadian home care clients.

### Comparison to similar works

To our knowledge, the original derivation and validation study for the DIVERT scale was the first to predict ED use among older home care clients using population-level data. The DIVERT scale performed similarly to other predictive models examining ED and hospital use among older adults [24–27]. Only one other study to date has attempted to predict ED use among home care clients. Jones et al. used a series of machine learning algorithms to predict ED use for an injurious fall within 6 months of a RAI-HC assessment [27]. A key difference between the DIVERT scale and prior work is that the DIVERT scale aims to inform program planning and preventative interventions prior to ED visitation by stratifying risk among subgroups of home care clients.

### Clinical and policy implications

Health systems (both public and private) are often limited by budgetary constraints, resulting in some clients not receiving adequate care; this is especially true of home care services, where many Canadian clients receive only partial care needs [28]. Limited home care services in Canada underscore the utility of systematically identifying clients who are at a greater risk of hospital use, to target enhanced risk assessment or preventative efforts.

The DIVERT scale provides real-time risk estimation and information that can be used to supplement decision making surrounding resource allocation and preventative interventions. For example, patients who score high-risk on the DIVERT scale (e.g., ≥ five or greater) with unstable cardiorespiratory symptoms are provided with a multifaceted and tailored service plan to meet their distinct needs [17, 29]. The DIVERT score can be further utilized among health care providers to ensure continuity of care and open communication regarding the risk of adverse events and health service use upon transitioning back to home care. As of recent, patients enrolled in the DIVERT trial are scheduled to receive nurse-led interventions focused on chronic disease management and patient education to facilitate patient-centred care.

Beyond its use for case finding, the DIVERT scale can be used to stratify or adjust organizational, regional and national level ED utilization metrics in home care settings. Our study demonstrated the external validity of the DIVERT scale and the utility that this prognostic tool is likely to have for home care clients across Canada. The RAI-HC is currently implemented as standard practice in many nations around the world, suggesting that knowledge translation efforts and clinical integration of the DIVERT scale are feasible in countries outside of Canada. Heterogeneity in the use of the DIVERT scale is likely to differ across health care systems, given the influence of funding models and health care standards.

### Limitations

The use of the DIVERT scale is limited to a predominantly frail population of community-dwelling older adults who receive home care services. The current study was limited to the person-level variables available in the RAI-HC assessment and could not capture all relevant determinants, particularly primary care utilization. Next, the predictive accuracy of the DIVERT scale was limited by the features of a complex adaptive system in home care delivery. Prior work has reported the difficulties in decision-making and predicting health outcomes in a complex adaptive system as they are more likely to feature non-linear interactions and a co-evolution of decisions and events [30–32]. Regardless of data complexity, the DIVERT scale achieved an AUC greater than 0.6 in all subgroup analyses in the original and external validation studies. This level of performance is similar to prior prognostic models aiming to predict ED and hospital use [24–26, 33]. However, we caution against precise comparisons of performance given that they cannot be compared within the study sample or against the same outcome measures. Further work is needed to understand what types and intensity of interventions are feasible and effective in the community. Future research should aim to replicate these findings in countries outside of Canada.

## Conclusion

Our study provided external validition for the DIVERT scale, further demonstrating that the tool can accurately predict ED use and hospitalization in Canadian home care clients. More specifically, our study demonstrated that the discriminative performance of this prognostic tool is consistent in a new cohort of clients from diverse regions and during a different time frame. Future research should aim to validate these findings in the United States and countries outside of North America, given that the implementation and uptake of the DIVERT scale in a new region is feasible.

## Data Availability

The data analyzed in this study are not publicly available due to privacy and confidentiality restrictions pertaining to person-level health information, which contains personal identifiers, in Canada; however, the data set creation plan and underlying analytic code are available from the corresponding author on reasonable request.

## Abbreviations

AUC: Area under the receiver operating characteristic curve
DIVERT: Detection of indicators and vulnerabilities for emergency room trips
ED: Emergency department
RAI-HC: Resident assessment instrument - home care
NACRS: National ambulatory care reporting system
